# Glutamine and Pediatric Intestinal Barrier Function: A Scoping Review of Clinical Evidence and Research Gaps

**DOI:** 10.3390/children13091231

**Published:** 2026-09-11

**Authors:** Dejan Dobrijević, Nataša Nastić, Kristian Pastor

**Affiliations:** 1 Institute for Children and Youth Health Care of Vojvodina, 21000 Novi Sad, Serbia; 2 Faculty of Medicine, University of Novi Sad, 21000 Novi Sad, Serbia; 3 Faculty of Technology Novi Sad, University of Novi Sad, 21000 Novi Sad, Serbia; 4 Khalifa University of Science and Technology, Abu Dhabi 127788, United Arab Emirates

**Keywords:** glutamine, intestinal barrier, intestinal permeability, pediatrics, necrotizing enterocolitis, short bowel syndrome, scoping review

## Abstract

**Highlights:**

**What are the main findings?**

- Pediatric evidence on glutamine and intestinal barrier function is heterogeneous and does not show a consistent overall benefit.
- Functional permeability is the most studied barrier domain, while mechanistic and microbial-translocation evidence remains limited.

**What are the implications of the main findings?**

- The observed effects of glutamine vary across clinical settings, intervention characteristics, and barrier assessment methods.
- Future pediatric studies should use standardized barrier outcomes and designs that isolate glutamine-specific effects.

**Abstract:**

**Background/Objectives:** Glutamine has been proposed as a nutritional modulator of intestinal epithelial integrity, but its effects on intestinal barrier function in children remain uncertain. This scoping review mapped the available human evidence on glutamine in relation to pediatric intestinal permeability, mucosal injury, microbial translocation, and intestinal adaptation. **Methods:** The review followed Joanna Briggs Institute methodology and PRISMA-ScR guidance. PubMed, Scopus, and Web of Science Core Collection were searched. Eligible studies included participants aged <18 years and assessed glutamine supplementation or endogenous glutamine status in relation to predefined barrier-related outcomes. Data were charted using a standardized form and synthesized descriptively according to clinical population, study design, glutamine exposure, and barrier domain. **Results:** Of 598 records identified, 434 unique records were screened. Eighteen publications representing 15 distinct or partially overlapping studies/cohorts were included. Functional intestinal permeability was the most frequently investigated domain. Findings were heterogeneous, with favorable, partial, transient, and null effects reported across different pediatric populations. Evidence for microbial translocation was limited and showed no clear benefit. Positive findings related to intestinal adaptation were mainly derived from uncontrolled or combined interventions, limiting attribution to glutamine. Direct mechanistic evidence in children was sparse, and no consistent major safety signal emerged, although the available safety evidence was limited. **Conclusions:** Current evidence does not support a uniform beneficial effect of glutamine on pediatric intestinal barrier function. Observed effects vary across populations, intervention characteristics, and outcome assessment methods.

## 1. Introduction

The intestinal epithelial barrier regulates the selective exchange of nutrients, water, and other luminal components while limiting the passage of potentially harmful microorganisms and antigens. Its disruption is relevant to several pediatric conditions, including prematurity and necrotizing enterocolitis (NEC), undernutrition and environmental enteropathy, gastrointestinal surgery, and intestinal failure. Barrier dysfunction may manifest through increased intestinal permeability, epithelial or mucosal injury, microbial translocation, or impaired structural and functional adaptation. These processes are particularly relevant during infancy and childhood, when intestinal maturation, nutritional requirements, and disease-related metabolic demands may substantially influence barrier integrity.

Glutamine has long attracted interest as a potential nutritional modulator of intestinal integrity. It serves as an important metabolic substrate for rapidly proliferating cells, including intestinal epithelial and immune cells, and participates in pathways related to cellular proliferation, nucleotide synthesis, antioxidant defense, and the response to physiological stress [1]. Experimental studies provide additional support for a direct epithelial effect. Glutamine availability has been shown to influence the expression and organization of tight-junction proteins, including claudin-1, occludin, and ZO-1 [2], while supplementation can attenuate experimentally induced changes in epithelial permeability and tight-junction integrity [3]. Glutamine has also been linked to cellular stress-response pathways involved in maintaining epithelial barrier function [4]. These mechanisms provide a strong biological rationale for glutamine supplementation, but evidence derived from experimental systems cannot establish its clinical effectiveness in children.

Pediatric studies have investigated glutamine in markedly different clinical settings and have produced inconsistent findings. In children at risk of environmental enteropathy, alanyl-glutamine has been evaluated in relation to functional permeability and inflammatory markers [1]. In preterm infants, similar enteral supplementation strategies have yielded both neutral and favorable effects on lactulose/mannitol permeability [5,6]. Observational evidence has additionally linked lower endogenous glutamine concentrations with NEC [7], further supporting biological interest without establishing a therapeutic effect of supplementation. Interpretation is complicated by differences in population, glutamine formulation, route, dose, treatment duration, co-interventions, and the methods used to define intestinal barrier dysfunction. Consequently, considering glutamine simply as an effective or ineffective pediatric nutritional intervention may obscure important differences between clinical contexts and barrier-related outcome domains.

Given the heterogeneity of the clinical populations, glutamine interventions, and barrier-related outcomes, a scoping review approach was considered appropriate to map the breadth and characteristics of the available evidence and to identify research gaps rather than to estimate a pooled treatment effect. Therefore, the aim of this scoping review was to map and characterize the available human evidence on glutamine in relation to intestinal barrier function and dysfunction in pediatric populations. Particular attention was given to functional intestinal permeability, epithelial and mucosal injury, microbial translocation and barrier failure, and intestinal adaptation. The review also sought to characterize the clinical contexts and approaches used to investigate these outcomes, examine the extent to which mechanistic evidence is available, and identify areas in which further pediatric research is needed.

## 2. Methods

### 2.1. Review Design

This scoping review was conducted in accordance with the Joanna Briggs Institute (JBI) methodological guidance for scoping reviews [8] and reported following the Preferred Reporting Items for Systematic Reviews and Meta-Analyses extension for Scoping Reviews (PRISMA-ScR) [9]. The review protocol was not prospectively registered or published.

### 2.2. Eligibility Criteria

Eligibility criteria were defined according to the Population–Concept–Context (PCC) framework [8]. The population comprised human participants from birth to <18 years of age, including neonates, preterm infants, infants, children, and adolescents. Mixed adult–pediatric studies were eligible only when pediatric data could be extracted separately.

The concept of interest was glutamine in relation to intestinal barrier function or dysfunction. Eligible interventions or exposures included free L-glutamine, alanyl-glutamine, and other glutamine-containing dipeptides administered enterally or parenterally. Studies evaluating endogenous glutamine status were eligible when glutamine concentrations were explicitly examined in relation to intestinal barrier injury or pathology. Combined interventions were retained when glutamine was an explicitly defined component.

Eligible barrier-related outcomes included: (i) functional intestinal permeability; (ii) structural or molecular epithelial integrity; (iii) epithelial or mucosal injury; (iv) microbial translocation or barrier failure; and (v) intestinal adaptation following intestinal resection or in short bowel syndrome. Studies reporting only microbiota composition, systemic inflammatory markers, growth, diarrhea, infection, sepsis, or mortality without an accompanying barrier-related outcome were excluded.

No restrictions were placed on pediatric clinical condition, geographical location, or healthcare setting. Randomized and non-randomized intervention studies and observational designs, including cohort, case–control, cross-sectional, case-series, and case-report designs, were eligible. Reviews, meta-analyses, editorials, commentaries, protocols, book chapters, and animal or in vitro studies were excluded from the formal evidence map. No language restriction was applied.

### 2.3. Information Sources and Search Strategy

PubMed, Scopus, and Web of Science Core Collection were searched from database inception to 9 July 2026. The search strategy combined three concept blocks relating to glutamine, pediatric populations, and intestinal barrier function or dysfunction. The PubMed strategy combined Medical Subject Headings (MeSH) with free-text terms, and equivalent strategies were adapted for Scopus and Web of Science according to their respective syntax and indexing structures.

Terms relating to intestinal barrier function encompassed intestinal permeability, epithelial and mucosal integrity, tight junctions, mucosal injury or mucositis, microbial or bacterial translocation, endotoxemia, intestinal adaptation, morphology, I-FABP, and zonulin. No publication-date, language, or document-type filters were applied. Complete database-specific search strategies are provided in Appendix A (Table A1). A supplementary targeted assessment of linked publications from potentially eligible pediatric trials was subsequently performed. No supplementary searching of reference lists, forward citations, clinical trial registries, or other sources was performed.

### 2.4. Study Selection

Records retrieved from the three databases were imported into Rayyan [10], where duplicates were identified and removed. Titles and abstracts were screened independently by two reviewers according to the predefined eligibility criteria, followed by independent full-text assessment of potentially eligible reports. Any disagreements between the reviewers were resolved through discussion and consensus. The selection process was documented using a PRISMA flow diagram.

Multiple publications originating from the same trial or overlapping participant cohort were linked rather than treated as independent studies when they reported distinct relevant analyses or outcomes.

### 2.5. Data Charting and Evidence Synthesis

Data were charted using a standardized Microsoft Excel form. Data were extracted by one reviewer and subsequently checked by a second reviewer for accuracy and completeness. Any discrepancies were resolved through discussion and consensus. Extracted variables included publication and study identifiers, country, study design, pediatric population, sample size and age, glutamine formulation, route, dose and duration, comparator and co-interventions, intestinal barrier domain and assessment method, principal barrier-related findings, mechanistic findings, relevant secondary outcomes, adverse events, and methodological limitations.

Barrier-related outcomes were classified as functional permeability, structural/molecular integrity, epithelial or mucosal injury, microbial translocation/barrier failure, or intestinal adaptation. Multiple reports from the same or overlapping study populations were linked during charting to avoid double counting.

The evidence was synthesized descriptively according to clinical population, glutamine exposure or intervention, study design, and barrier domain. During descriptive synthesis, the interpretive labels were assigned according to the direction and consistency of the reported barrier-related findings. Favorable findings indicated an overall beneficial barrier signal, whereas mixed, limited, or transient findings indicated effects restricted to selected endpoints, subgroups, or time points. These labels were descriptive and were not intended as formal effect or certainty-of-evidence ratings. Given the heterogeneity of the included evidence and the mapping objective of the review, no meta-analysis was performed. Methodological characteristics relevant to interpretation, including study design, sample size, comparator, randomization, blinding, co-interventions, and barrier assessment methods, were considered in the narrative synthesis. Consistent with the scoping-review objective, no formal critical appraisal, risk-of-bias assessment, or certainty-of-evidence assessment of the included sources was performed.

## 3. Results

### 3.1. Study Selection

**Figure 1:**
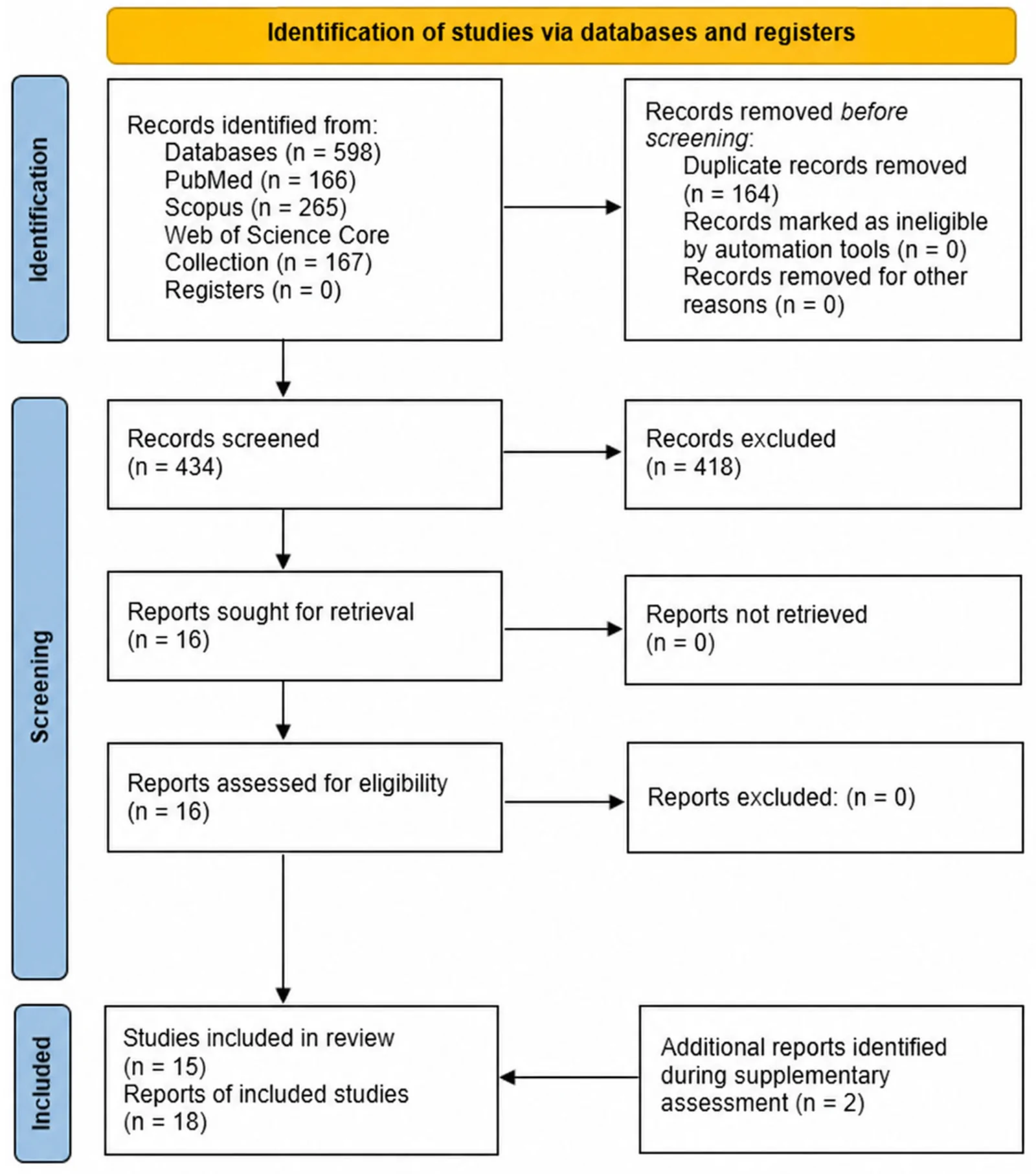
PRISMA-ScR flow diagram of study selection.

The database searches identified 598 records, including 166 from PubMed, 265 from Scopus, and 167 from Web of Science Core Collection. After removal of 164 duplicate records, 434 unique records underwent title and abstract screening. Of these, 418 were excluded because they did not meet the predefined eligibility criteria. The full texts of the remaining 16 publications were retrieved and assessed for eligibility, and all 16 met the inclusion criteria and were included in the scoping review. During supplementary assessment, two additional eligible reports from the same randomized pediatric Crohn’s disease trial were identified and included. The final review therefore included 18 publications representing 15 studies or cohorts (Figure 1). Specifically, Lima et al. [11] and Mitter et al. [12] originated from the same parent trial (NCT00133406), while the reports by Guo et al. [13,14] represented an overlapping and subsequently expanded pediatric short bowel syndrome cohort. These publications were retained separately because they provided distinct relevant data, but their participant populations were not treated as independent evidence. The two reports by Akobeng et al. originated from the same randomized pediatric Crohn’s disease trial and were likewise treated as one study-level evidence source.

### 3.2. Characteristics of the Included Evidence

The 15 studies were published between 2000 and 2026 and were conducted in nine countries: Brazil, the Netherlands, Greece, The Gambia, the United Kingdom, the United States, China, Japan, and Indonesia. The evidence covered a broad pediatric age range, from extremely preterm neonates to adolescents aged 17 years. Clinical contexts included malnutrition and environmental enteropathy, prematurity and very low birth weight, necrotizing enterocolitis (NEC), gastrointestinal surgery, short bowel syndrome (SBS), active Crohn’s disease, high-output stoma, and children with altered markers of intestinal mucosal integrity.

Twelve of the 15 studies contained a randomized intervention component, although the evidence base also included a prospective observational cohort, retrospective uncontrolled studies, and a single case report. Most interventional studies evaluated free L-glutamine, whereas two studies investigated alanyl-glutamine [1,15]. Becker et al. [7] evaluated endogenous serum glutamine rather than supplementation. Glutamine was administered predominantly by the enteral or oral route. Albers et al. [16] used parenteral glutamine, while Bishay et al. [17] combined parenteral and enteral administration. Doses, treatment duration, and follow-up varied considerably between studies. Several interventions also included other nutritional or therapeutic components, notably zinc and vitamin A [11,12], growth hormone and enteral nutrition [13,14], partially hydrolyzed guar gum [18], or zinc, fiber, and prebiotics [19], limiting attribution of observed effects specifically to glutamine (Table 1). Accordingly, the evidence base included studies in which a glutamine-specific intervention effect could be assessed, combined interventions in which the independent contribution of glutamine could not be isolated, and one observational study evaluating endogenous glutamine status. Findings from uncontrolled studies and the single case report were interpreted more cautiously and were not given the same evidential weight as findings from randomized controlled trials.

### 3.3. Functional Intestinal Permeability

Functional intestinal permeability was the most frequently investigated barrier domain, evaluated in nine studies and reported across 11 publications. Most studies used urinary lactulose-to-mannitol (L/M) testing, whereas Albers et al. [16] used the lactulose-to-rhamnose ratio. At the study level, three studies showed a favorable permeability signal, two showed mixed or limited effects, and four showed no overall benefit or a possible unfavorable trend.

Among children with malnutrition or environmental enteropathy, findings were heterogeneous. Lima et al. [20] reported a marked reduction in the L/M ratio after 10 days of L-glutamine supplementation, from 0.3129 ± 0.1025 to 0.1044 ± 0.0175 (*p* = 0.01). No significant within-group change occurred in either the nonsupplemented or glycine groups, although interpretation was complicated by the use of an earlier nonsupplemented comparison cohort and by lower post-treatment L/M values in the glycine group than in nonsupplemented controls.

In a subsequent randomized trial, Lima et al. [15] found that 10 days of high-dose alanyl-glutamine significantly reduced urinary lactulose excretion from a median of 0.222% to 0.082% and mannitol excretion from 6.156% to 2.756%. However, the L/M ratio itself did not significantly improve, making the permeability effect difficult to interpret as an unequivocal improvement in barrier function.

The NCT00133406 trial provided additional evidence concerning glutamine and permeability in Brazilian children. In the primary report, Lima et al. [11] found significantly lower urinary lactulose and mannitol excretion after glutamine compared with placebo–glycine, while the combination of glutamine, zinc, and vitamin A produced additional changes in the L/M ratio. The authors therefore reported improvement in barrier-related measures, although interpretation of the combined intervention was limited by co-administration of the other gut-trophic nutrients.

The companion analysis by Mitter et al. [12] investigated APOE4 as a potential modifier of responses to nutritional supplementation. Although modest decreases in L/M occurred among glutamine-treated subgroups, APOE4 carriage did not significantly modify the glutamine-associated permeability response. The genotype-related permeability effect was observed primarily with vitamin A rather than glutamine.

In the more recent IMAGINE dose–response trial, Moore et al. [1] found that the 12 g/day alanyl-glutamine group showed a reduction in median urinary lactulose excretion from 0.193% at baseline to 0.107% after 10 days (*p* = 0.05). However, the effect was no longer present at day 30, and neither the L/M ratio nor mannitol excretion showed a corresponding significant improvement. Thus, the barrier-related effect was transient and endpoint-specific rather than a consistent improvement in overall intestinal permeability.

In contrast, prolonged supplementation did not improve permeability in growth-faltering Gambian infants. Williams et al. [21] reported final geometric mean L/M ratios of 0.29 in the glutamine group and 0.26 in controls. Repeated-measures analysis indicated a borderline higher L/M ratio with glutamine, although individual monthly comparisons and the time-by-treatment interaction were not significant. Glutamine also failed to improve growth, morbidity, or systemic inflammatory and immunological measures. In children with active Crohn’s disease, Akobeng et al. [23,24] compared a glutamine-enriched polymeric diet with a standard low-glutamine polymeric diet. After four weeks, the mean L/M ratio decreased from 0.103 to 0.037 in the standard-diet group and from 0.068 to 0.043 in the glutamine-enriched group, with no significant between-group difference in the change in permeability (*p* = 0.239). Thus, glutamine enrichment did not provide an additional permeability benefit.

The two randomized trials in premature infants yielded contrasting findings. Van den Berg et al. [5] observed the expected postnatal decline in the L/M ratio in both glutamine and control groups but found no additional effect of glutamine supplementation (*p* = 0.78). The authors therefore concluded that enteral glutamine did not accelerate the physiological improvement in intestinal permeability during the first month of life.

Conversely, Sevastiadou et al. [6] reported significantly lower L/M ratios in glutamine-supplemented preterm neonates than in controls at both day 7 (0.539 vs. 1.179; *p* = 0.004) and day 30 (0.637 vs. 1.002; *p* = 0.001). Lactulose recovery was also lower, and the glutamine group had lower reported incidences of NEC and septicemia during follow-up.

Finally, parenteral glutamine did not improve postoperative permeability in newborns and infants undergoing major digestive-tract surgery. Albers et al. [16] found no significant difference between glutamine-supplemented and standard parenteral nutrition in the lactulose:rhamnose ratio over four postoperative weeks. No significant benefits were found for nitrogen balance, septic episodes, hospital stay, or other major clinical outcomes.

The permeability studies did not demonstrate a uniform effect of glutamine across pediatric populations. Favorable findings were present in some malnourished children and in one preterm neonatal trial, whereas other studies showed transient or endpoint-specific effects, no improvement, or a possible borderline unfavorable permeability trend.

### 3.4. Microbial Translocation and Barrier Failure

Direct assessment of microbial translocation was limited to one randomized trial. Bishay et al. [17] investigated surgical infants requiring parenteral nutrition and defined microbial invasion using a composite endpoint comprising conventional blood culture, microbial DNA detected by PCR, plasma endotoxin, and lipopolysaccharide-binding protein. After five days, microbial invasion occurred in 9 of 31 control infants and 8 of 29 glutamine-treated infants, corresponding to an adjusted odds ratio of 0.83 (95% CI 0.24–2.86; *p* = 0.77). No significant benefit was observed for the individual components of the composite endpoint or for microbial invasion during the overall study period.

Despite the null primary outcome, glutamine supplementation was associated with improved recovery of monocyte HLA-DR expression. An exploratory subgroup analysis also suggested a possible reduction in microbial invasion among infants with low baseline HLA-DR expression. These findings indicated a possible immunological effect but did not demonstrate an overall reduction in microbial translocation [17].

### 3.5. Mucosal Injury, Inflammation, and Endogenous Glutamine Status

Becker et al. [7] evaluated endogenous amino-acid status prospectively in 45 premature infants, 13 of whom subsequently developed NEC. Serum glutamine concentrations were 37–57% lower in infants who developed NEC on days 7, 14, and 21. Reduced glutamine and arginine concentrations were detectable before clinical NEC developed, suggesting that altered amino-acid status preceded rather than merely accompanied the intestinal disease. These observational findings, however, demonstrate association and do not establish that glutamine supplementation would prevent NEC.

Kadim et al. [19] evaluated fecal calprotectin and α1-antitrypsin in Indonesian children aged 1–3 years receiving either a standard formula or a test formula containing glutamine together with additional zinc, fiber, and prebiotics. No significant overall between-group differences in either marker were observed after six or ten months. In a post hoc subgroup with normal fecal calprotectin at baseline, a greater reduction was observed with the supplemented formula at six months, but this difference was not sustained at month 10 and was not observed in children with elevated baseline calprotectin.

### 3.6. Intestinal Adaptation and Structural or Absorptive Outcomes

Evidence concerning intestinal adaptation included an overlapping pediatric SBS cohort reported in two publications, a single case report with direct intestinal histology, and a small randomized pilot study in surgical infants.

Guo et al. [13] retrospectively evaluated 16 children with SBS undergoing an intestinal rehabilitation program combining growth hormone, glutamine, and enteral nutrition. Nitrogen absorptive capacity increased from 51.59 ± 5.36% before treatment to 84.69 ± 1.17% after treatment (*p* < 0.001), accompanied by improvements in nutritional indices and parenteral-nutrition independence. Because glutamine was given as one component of a multimodal rehabilitation program and the study lacked a control group, its independent contribution could not be determined.

The earlier report from the overlapping cohort by Guo et al. [14] similarly showed an increase in nitrogen absorption from 65.48 ± 6.97% to 86.98 ± 0.81% (*p* = 0.038) and an increase in D-xylose absorption from 1.94 ± 0.60 to 3.22 ± 0.22 mmol/L (*p* = 0.043). Six of the seven children were subsequently weaned from parenteral nutrition. As in the later report, glutamine was administered together with growth hormone, enteral nutrition, and dietary modification.

Direct structural evidence was reported by Kawaguchi et al. [18] in a single extremely low-birth-weight infant with NEC, high-output stoma, and villous atrophy. Following combined supplementation with glutamine and partially hydrolyzed guar gum, median villus height increased from 144 to 229 μm (*p* = 0.03), while crypt depth and villus width were unchanged. Stoma output decreased and enteral feeding tolerance increased during the same period. Although this study provided direct histological evidence of intestinal adaptation, the single-case design, concurrent PHGG supplementation, and postoperative recovery prevented attribution of the changes specifically to glutamine.

In contrast, the randomized pilot study by Duggan et al. [22] did not identify a benefit of enteral glutamine in infants following surgery for gastrointestinal disease. No significant improvement was observed in intestinal nutrient absorption, duration of parenteral nutrition, progression to enteral feeding, growth, or infection. Detailed nutrient-balance measurements were available for only seven infants, and the study was underpowered because of its small sample size and high clinical variability.

A summary of the intestinal barrier assessment methods and principal barrier-related findings across the included publications is provided in Table 2.

### 3.7. Mechanistic and Safety Findings

Direct mechanistic assessment in the included pediatric studies was limited. Moore et al. [1] reported a reduction in fecal lactoferrin with the lowest alanyl-glutamine dose, but no consistent changes were found in α1-antitrypsin, myeloperoxidase, neopterin, Reg-1β, fecal cytokines, or urinary metabolomic profiles.

Mitter et al. [12] explored APOE4 as a host-related modifier, but no clear glutamine-specific genotype effect on intestinal permeability was demonstrated.

Bishay et al. [17] demonstrated increased monocyte HLA-DR expression during glutamine supplementation despite the absence of an effect on microbial translocation, providing evidence of an immunological response that did not translate into improvement in the primary barrier-failure endpoint. Becker et al. [7] provided a temporal association between reduced endogenous glutamine and subsequent NEC but did not directly investigate epithelial mechanisms. The villous elongation observed by Kawaguchi et al. [18] represented the only direct longitudinal histological evidence of mucosal structural change, although it occurred during combined glutamine and PHGG supplementation.

No consistent major safety signal attributable to glutamine emerged across the included studies; however, the studies were generally small and were not primarily designed to assess safety, limiting firm conclusions regarding pediatric safety. Moore et al. [1] reported no difference in adverse events between treatment groups and no serious adverse events attributable to supplementation. Lima et al. [15] described high-dose alanyl-glutamine as well tolerated. Albers et al. [16] observed slightly higher serum ammonia concentrations with parenteral glutamine (44 vs. 34 μmol/L; *p* = 0.008), but the difference was not considered clinically relevant. Bishay et al. [17] reported no study-related serious adverse events, while the minor adverse events reported in the SBS rehabilitation studies were primarily related to enteral feeding or growth hormone rather than clearly to glutamine [13,14].

## 4. Discussion

The available pediatric evidence does not indicate a consistent effect of glutamine on intestinal barrier function. Instead, the findings vary substantially across clinical settings. Favorable findings were observed in some studies of malnutrition and prematurity [6,11,20], while other studies of malnutrition or environmental enteropathy showed mixed, limited, or transient effects [1,15]. In contrast, several controlled studies in preterm, surgical, growth-faltering, or Crohn’s disease populations found no overall benefit [5,16,21,23,24]. Evidence for microbial translocation was also negative [17], while favorable findings concerning intestinal adaptation were derived mainly from combined interventions in which glutamine could not be separated from growth hormone, enteral nutrition, fiber, or spontaneous adaptation [13,14,18]. Thus, the central finding of this review is not simply that results are heterogeneous, but that the clinical setting and the way barrier function is measured appear critical to how the effect of glutamine is interpreted. In interpreting the overall evidence, greater emphasis was therefore placed on controlled randomized findings, whereas favorable results from uncontrolled studies and the single case report were considered supportive but insufficient to establish a glutamine-specific effect.

This is particularly evident for functional permeability, the most frequently investigated outcome. Most studies relied on lactulose-based dual-sugar tests, but changes in individual sugar recovery were not always accompanied by corresponding changes in the permeability ratio [1,15]. Lactulose primarily reflects paracellular passage, whereas the denominator also incorporates absorptive characteristics; consequently, simultaneous changes in both sugars can complicate interpretation of the ratio. This may partly explain why some pediatric studies reported improvement in individual permeability components without demonstrating a clear overall effect on L/M. Differences in dose, duration, baseline barrier impairment, and timing of measurement provide additional sources of heterogeneity. The transient effect observed in the dose–response trial by Moore et al. [1], for example, also raises the possibility that short-term changes in permeability do not necessarily indicate persistent restoration of barrier function.

Barrier-related assessment methods should not be considered interchangeable because they capture different biological processes. Dual-sugar tests primarily assess functional paracellular permeability, whereas circulating I-FABP reflects enterocyte injury, citrulline more closely reflects functional enterocyte mass, and LBP and sCD14 provide indirect information related to microbial translocation or the host response to microbial products. Zonulin has also been proposed as a marker of barrier regulation, although concerns regarding assay specificity limit its interpretation, while fecal calprotectin primarily reflects intestinal inflammation rather than permeability itself. These approaches should therefore be viewed as complementary rather than equivalent measures of intestinal barrier dysfunction [25].

Experimental evidence nevertheless provides a plausible biological basis for a barrier-protective effect of glutamine. Glutamine availability has been linked to the expression and localization of tight-junction proteins, including claudin-1, occludin, and ZO-1 [2,3], and to cellular stress responses involved in epithelial protection [4]. Adult studies further indicate that glutamine can reduce increased permeability under some conditions, including experimentally induced intestinal injury and postinfectious intestinal hyperpermeability [26,27,28], whereas other clinical studies have shown no improvement [29]. The inconsistency seen in children therefore does not necessarily contradict the proposed epithelial mechanisms. Rather, it suggests that biological activity does not uniformly translate into a measurable clinical barrier effect. Notably, none of the pediatric intervention studies directly demonstrated that changes in tight-junction proteins or related epithelial pathways mediated the observed clinical findings.

The contrasting findings in preterm infants illustrate this problem especially clearly. Similar enteral doses and supplementation periods produced a positive permeability signal in one trial and no additional effect beyond normal postnatal maturation in another [5,6]. The observational association between low endogenous glutamine and subsequent NEC [7] provides biological context but cannot demonstrate that supplementation prevents intestinal injury. Together, these findings indicate that glutamine deficiency, permeability abnormalities, and clinical benefit from glutamine supplementation should not be treated as equivalent concepts.

The evidence from gastrointestinal surgery and SBS requires similar caution. The uncontrolled pediatric rehabilitation studies showed improved absorption and reduced dependence on parenteral nutrition [13,14], but glutamine was only one component of an intensive rehabilitation strategy. The same limitation applies to the histological improvement reported with combined glutamine and PHGG supplementation [18]. Adult SBS studies have likewise produced conflicting results when glutamine has been combined with growth hormone and dietary modification [30,31]. These findings make it difficult to determine whether glutamine contributes independently to adaptation, potentiates another intervention, or has little additional effect beyond nutritional rehabilitation and natural adaptation. The negative randomized pilot study in surgical infants [22] further argues against assuming that the trophic role of glutamine demonstrated experimentally necessarily produces clinically relevant adaptation in pediatric patients.

The mechanistic evidence identified within the pediatric literature was notably limited. Changes in inflammatory markers, monocyte HLA-DR expression, endogenous amino acid concentrations, and villus morphology provide potentially relevant observations [1,7,17,18], but they represent different biological levels and cannot be combined into a single mechanism. Future studies would be more informative if functional permeability were evaluated alongside epithelial injury markers and, where feasible, molecular or histological measures of junctional integrity. This would allow investigators to distinguish a true epithelial effect from secondary changes associated with inflammation, nutritional recovery, or disease resolution.

Glutamine was generally well tolerated in the included studies, but the evidence base is insufficient to draw firm conclusions regarding pediatric safety or to define an optimal pediatric dose, formulation, route, or duration. In the Akobeng trial, two participants in the glutamine-enriched diet group were withdrawn because of intolerance to the diet [23,24]. More importantly, future research may benefit less from additional small heterogeneous supplementation trials and more from well-defined studies in populations with documented barrier dysfunction at baseline. Such trials should prespecify a primary barrier endpoint, distinguish individual sugar recoveries from permeability ratios, standardize the timing of measurement, and minimize or explicitly account for co-interventions. Dose–response studies and direct comparisons between free glutamine and glutamine dipeptides would also address questions that remain largely unresolved.

Several limitations of this scoping review should be considered. The broad inclusion criteria were intended to map the pediatric literature but resulted in substantial clinical and methodological heterogeneity. Some included publications represented the same or overlapping study populations and were therefore linked rather than treated as independent evidence. In accordance with the scoping-review design, no formal risk-of-bias or certainty-of-evidence assessment was performed, and the findings should therefore not be interpreted as a quantitative estimate of treatment efficacy. Experimental and adult studies were used only to interpret the pediatric evidence and were not part of the formal evidence map. In addition, the search was limited to three bibliographic databases and did not include supplementary citation searching or clinical trial registries.

Current evidence supports a biologically plausible role for glutamine in intestinal epithelial maintenance but does not support a general conclusion that glutamine supplementation improves intestinal barrier function in children. The observed heterogeneity may reflect differences in the underlying condition, baseline barrier impairment, intervention characteristics, and the outcome used to assess barrier function. The main research priority is therefore not simply to determine whether glutamine “works”, but to establish in which pediatric populations, under which conditions, and through which measurable mechanisms it may provide clinically relevant benefit.

## 5. Conclusions

The available pediatric evidence does not demonstrate a consistent benefit of glutamine supplementation on intestinal barrier function. Favorable effects were reported in some settings, whereas other controlled studies showed transient, endpoint-specific, or no measurable benefit. Interpretation remains limited by heterogeneity in populations, interventions, barrier assessment methods, and co-interventions. Overall, current evidence supports biological plausibility but does not establish a general barrier-protective effect of glutamine in children.

## Figures and Tables

**Table 1:** Characteristics of the included studies evaluating glutamine and intestinal barrier-related outcomes in pediatric populations.

Study	Country; Design	Population	Sample Size; Age	Glutamine Intervention/Exposure	Comparator/Co-Interventions	Barrier Domain
Moore et al., 2020 [1]	Brazil; double-blind dose–response RCT	Children at risk of environmental enteropathy/undernutrition	n = 140; 2–60 months	Ala-Gln, 3/6/12 g/day orally for 10 days	Glycine	Functional permeability
Lima et al., 2007 [15]	Brazil; double-blind RCT	Undernourished community children	n = 107; 7.9–82.2 months	Ala-Gln, 24 g/day orally for 10 days	Glycine	Functional permeability
Lima et al., 2005 [20]	Brazil; controlled study; randomized	Hospitalized malnourished children	n = 80; 2–60 months	L-Gln, 16.2 g/day for 10 days	Glycine/nonsupplemented formula	Functional permeability
Lima et al., 2014 [11]; Mitter et al., 2012 [12]	Brazil; randomized factorial trial	Children with undernutrition/high enteric-disease burden	Parent trial n = 314; ~5 months–9 years	L-Gln, ~16 g/day for 10 days	Glycine; ±zinc/vitamin A	Functional permeability
van den Berg et al., 2006 [5]	Netherlands; double-blind RCT	VLBW/preterm infants	n = 102; GA ~29 weeks	L-Gln, ≤0.3 g/kg/day, days 3–30	L-alanine	Functional permeability
Sevastiadou et al., 2011 [6]	Greece; double-blind RCT	Preterm neonates	n = 101; GA <34 weeks	L-Gln, 0.3 g/kg/day, days 3–30	Isocaloric placebo	Functional permeability
Albers et al., 2005 [16]	Netherlands; double-blind RCT	Infants after GI surgery	n = 80; <2 years	Parenteral L-Gln, 0.4 g/kg/day	Standard PN	Functional permeability
Williams et al., 2007 [21]	The Gambia; double-blind RCT	Growth-faltering infants	n = 93; 4–11 months	L-Gln, 0.5 g/kg/day for 5–6 months	Amino-acid placebo	Functional permeability
Bishay et al., 2020 [17]	UK; double-blind RCT	Surgical infants requiring PN	n = 60 analyzed; <3 months	Parenteral + enteral Gln, 0.4 g/kg/day	Isonitrogenous control	Microbial translocation
Becker et al., 2000 [7]	USA; prospective cohort	Premature infants followed for NEC	n = 45; GA < 35 weeks	Endogenous serum glutamine	—	Intestinal injury/NEC
Guo et al., 2016 [13]; Guo et al., 2012 [14]	China; retrospective cohort	Pediatric SBS	n = 16 expanded cohort; 2–17 years	L-Gln, 0.45 g/kg/day	GH + EN	Intestinal adaptation
Kawaguchi et al., 2026 [18]	Japan; case report	VLBW infant with NEC/high-output stoma	n = 1; GA 26 + 3 weeks	L-Gln, 0.3 g/kg/day	PHGG	Structural integrity/adaptation
Kadim et al., 2020 [19]	Indonesia; randomized trial	Presumed healthy children	n = 200; 1–3 years	L-Gln, 0.7 g/day	Zinc + fiber + prebiotics	Mucosal integrity
Duggan et al., 2004 [22]	USA; pilot RCT	Infants after GI surgery	n = 20; <12 months	L-Gln, target 0.4 g/kg/day	Amino-acid placebo	Intestinal adaptation
Akobeng et al., 2000 [23,24]	UK; double-blind RCT	Children with active Crohn’s disease	n = 18; 6–16 years	Glutamine-enriched polymeric diet; 42% of amino-acid composition; 4 weeks	Standard low-glutamine polymeric diet; 4% of amino-acid composition	Functional permeability

Abbreviations: EN, enteral nutrition; GA, gestational age; GI, gastrointestinal; NEC, necrotizing enterocolitis; PHGG, partially hydrolyzed guar gum; PN, parenteral nutrition; RCT, randomized controlled trial; SBS, short bowel syndrome; VLBW, very-low-birth-weight. **Note**: Lima et al. (2014) [11] and Mitter et al. (2012) [12] represent reports from the same parent trial (NCT00133406). Guo et al. (2012) [14] and Guo et al. (2016) [13] represent overlapping/expanded cohorts and were therefore treated as one study-level evidence source. Akobeng et al. (2000) [23,24] reported different outcomes from the same randomized pediatric Crohn’s disease trial and were treated as one study-level evidence source.

**Table 2:** Intestinal barrier-related assessments and principal findings of the included publications.

Study	Barrier Assessment	Assessment Timing	Main Barrier-Related Finding	Interpretation
Moore et al., 2020 [1]	Urinary lactulose/mannitol; individual sugar recovery	Baseline, day 10, day 30	At 12 g/day, lactulose decreased from 0.193% to 0.107% at day 10 (*p* = 0.05), but the effect was not sustained; L/M ratio did not improve	Mixed/transient benefit
Lima et al., 2007 [15]	Urinary lactulose/mannitol	Days 1 and 10	Lactulose and mannitol excretion decreased significantly with Ala-Gln, but L/M ratio did not change significantly	Mixed/limited benefit
Lima et al., 2005 [20]	Urinary lactulose/mannitol	Before and after 10 days	L/M decreased from 0.3129 ± 0.1025 to 0.1044 ± 0.0175 (*p* = 0.01) with glutamine	Favorable, with comparator limitations
Lima et al., 2014 [11]	Urinary lactulose/mannitol and individual sugar recovery	Baseline and 4 months	Glutamine reduced lactulose and mannitol excretion versus placebo; combined glutamine + zinc + vitamin A also affected L/M	Favorable, endpoint-specific
Mitter et al., 2012 [12]	Urinary lactulose/mannitol; APOE4 analysis	Baseline and 4 months	L/M decreased modestly in glutamine subgroups, but APOE4 did not significantly modify the glutamine response	No clear independent effect in secondary analysis
van den Berg et al., 2006 [5]	Urinary lactulose/mannitol	Baseline, days 7, 14, 30	Normal postnatal decline in L/M occurred in both groups; no glutamine effect (*p* = 0.78)	No effect
Sevastiadou et al., 2011 [6]	Urinary lactulose/mannitol	Days 2, 7, 30	Lower L/M with glutamine at day 7 (0.539 vs. 1.179; *p* = 0.004) and day 30 (0.637 vs. 1.002; *p* = 0.001)	Favorable
Albers et al., 2005 [16]	Urinary lactulose/rhamnose	Postoperative days 5, 12, 19, 26	No significant difference between glutamine and standard PN over time (*p* = 0.11)	No effect
Williams et al., 2007 [21]	Urinary lactulose/mannitol	Monthly during 5–6 months	No improvement; final L/M 0.29 vs. 0.26, with borderline higher overall L/M in glutamine group	No benefit; possible borderline adverse trend
Bishay et al., 2020 [17]	Blood culture, microbial DNA PCR, endotoxin, LBP	Baseline, day 5 and subsequent clinical time points	Microbial invasion: 8/29 glutamine vs. 9/31 control; adjusted OR 0.83 (95% CI 0.24–2.86; *p* = 0.77)	No effect
Becker et al., 2000 [7]	Serum glutamine and amino-acid profile; NEC occurrence	Days 3, 7, 14, 21	Serum glutamine was 37–57% lower in infants who developed NEC and was reduced before clinical NEC	Observational association
Guo et al., 2016 [13]	Nitrogen absorptive rate; PN dependence	Pre-/post-rehabilitation and long-term follow-up	Nitrogen absorption increased from 51.59 ± 5.36% to 84.69 ± 1.17% (*p* < 0.001)	Favorable; glutamine effect not isolatable
Guo et al., 2012 [14]	Nitrogen absorption; D-xylose absorption	Pre-/post-treatment and follow-up	Nitrogen absorption increased from 65.48 ± 6.97% to 86.98 ± 0.81% (*p* = 0.038); D-xylose also increased	Favorable; glutamine effect not isolatable
Kawaguchi et al., 2026 [18]	Jejunal histology; villus height; stoma output	Before supplementation and after 4 weeks	Villus height increased from 144 to 229 μm (*p* = 0.03); stoma output decreased	Favorable; attribution very limited
Kadim et al., 2020 [19]	Fecal calprotectin and α1-antitrypsin	Baseline, months 6 and 10	No overall between-group benefit; transient post hoc FC reduction at 6 months in children with normal baseline FC	No overall benefit
Duggan et al., 2004 [22]	Nutrient/energy balance; absorptive function	During study and at study completion	No improvement in nutrient absorption, PN duration, or progression to enteral feeding	No effect
Akobeng et al., 2000 [23,24]	Urinary lactulose/mannitol ratio	Baseline and 4 weeks	L/M decreased in both groups; no significant between-group difference in change (*p* = 0.239)	No effect

Abbreviations: Ala-Gln, alanyl-glutamine; FC, fecal calprotectin; LBP, lipopolysaccharide-binding protein; L/M, lactulose-to-mannitol ratio; NEC, necrotizing enterocolitis; PN, parenteral nutrition. Note: Findings from combined interventions were classified according to the overall barrier-related outcome, but the independent contribution of glutamine could not be determined.

## Data Availability

No new primary data were generated in this study. All data analyzed in this scoping review were obtained from the published studies included in the review.

## Abbreviations

The following abbreviations are used in this manuscript:

Ala-Gln	Alanyl-glutamine
APOE4	Apolipoprotein E4
CI	Confidence interval
EN	Enteral nutrition
FC	Fecal calprotectin
GA	Gestational age
GH	Growth hormone
GI	Gastrointestinal
Gln	Glutamine
HLA-DR	Human leukocyte antigen-DR
I-FABP	Intestinal fatty acid-binding protein
JBI	Joanna Briggs Institute
LBP	Lipopolysaccharide-binding protein
L/M	Lactulose-to-mannitol ratio
MeSH	Medical Subject Headings
NEC	Necrotizing enterocolitis
OR	Odds ratio
PCC	Population–Concept–Context
PCR	Polymerase chain reaction
PHGG	Partially hydrolyzed guar gum
PN	Parenteral nutrition
PRISMA-ScR	Preferred Reporting Items for Systematic Reviews and Meta-Analyses Extension for Scoping Reviews
RCT	Randomized controlled trial
SBS	Short bowel syndrome
VLBW	Very-low-birth-weight

## Appendix A

**Table A1:** Complete database-specific search strategies used to identify studies on glutamine and intestinal barrier function in pediatric populations.

Database	Search Date	Complete Search Strategy	Filters Applied	Records Retrieved
PubMed	9 July 2026	(“Glutamine”[Mesh] OR glutamine[tiab] OR “L-glutamine”[tiab] OR “alanyl-glutamine”[tiab] OR alanylglutamine[tiab] OR “Ala-Gln”[tiab] OR “glutamine dipeptide”[tiab] OR “glutamine-containing dipeptide”[tiab]) AND (“Infant”[Mesh] OR “Child”[Mesh] OR “Adolescent”[Mesh] OR “Infant, Newborn”[Mesh] OR “Infant, Premature”[Mesh] OR pediatric[tiab] OR paediatric[tiab] OR child[tiab] OR infant[tiab] OR neonat[tiab] OR newborn[tiab] OR preterm[tiab] OR premature[tiab] OR adolescen[tiab]) AND (“Intestinal Barrier Function”[Mesh] OR “Intestinal Mucosa”[Mesh] OR “intestinal barrier”[tiab] OR “gut barrier”[tiab] OR “gastrointestinal barrier”[tiab] OR “intestinal permeability”[tiab] OR “gut permeability”[tiab] OR “gastrointestinal permeability”[tiab] OR “mucosal permeability”[tiab] OR “intestinal mucosal barrier”[tiab] OR “gut mucosal barrier”[tiab] OR “mucosal barrier”[tiab] OR “intestinal epithelial barrier”[tiab] OR “gut epithelial barrier”[tiab] OR “epithelial barrier”[tiab] OR “mucosal integrity”[tiab] OR “intestinal integrity”[tiab] OR “gut integrity”[tiab] OR “epithelial integrity”[tiab] OR “tight junction”[tiab] OR “intestinal mucosal injury”[tiab] OR “intestinal epithelial injury”[tiab] OR “intestinal injury”[tiab] OR “gastrointestinal mucositis”[tiab] OR “intestinal mucositis”[tiab] OR “bacterial translocation”[tiab] OR endotoxemia[tiab] OR endotoxaemia[tiab] OR “intestinal adaptation”[tiab] OR “intestinal morphology”[tiab] OR “mucosal morphology”[tiab] OR “I-FABP”[tiab] OR “intestinal fatty acid binding protein”[tiab] OR zonulin[tiab])	None	166
Scopus	9 July 2026	TITLE-ABS-KEY((glutamine OR “L-glutamine” OR “alanyl-glutamine” OR alanylglutamine OR “Ala-Gln” OR “glutamine dipeptide” OR “glutamine-containing dipeptide”) AND (pediatric OR paediatric OR child OR infant OR neonat OR newborn OR preterm OR premature OR adolescen) AND (“intestinal barrier” OR “gut barrier” OR “gastrointestinal barrier” OR “intestinal permeability” OR “gut permeability” OR “gastrointestinal permeability” OR “mucosal permeability” OR “intestinal mucosal barrier” OR “gut mucosal barrier” OR “mucosal barrier” OR “intestinal epithelial barrier” OR “gut epithelial barrier” OR “epithelial barrier” OR “mucosal integrity” OR “intestinal integrity” OR “gut integrity” OR “epithelial integrity” OR “tight junction” OR “intestinal mucosal injury” OR “intestinal epithelial injury” OR “intestinal injury” OR “gastrointestinal mucositis” OR “intestinal mucositis” OR “bacterial translocation” OR endotoxemia OR endotoxaemia OR “intestinal adaptation” OR “intestinal morphology” OR “mucosal morphology” OR “I-FABP” OR “intestinal fatty acid binding protein” OR zonulin))	None	265
Web of Science Core Collection	9 July 2026	TS = (glutamine OR “L-glutamine” OR “alanyl-glutamine” OR alanylglutamine OR “Ala-Gln” OR “glutamine dipeptide” OR “glutamine-containing dipeptide”) AND TS = (pediatric OR paediatric OR child OR infant OR neonat OR newborn OR preterm OR premature OR adolescen) AND TS = (“intestinal barrier” OR “gut barrier” OR “gastrointestinal barrier” OR “intestinal permeability” OR “gut permeability” OR “gastrointestinal permeability” OR “mucosal permeability” OR “intestinal mucosal barrier” OR “gut mucosal barrier” OR “mucosal barrier” OR “intestinal epithelial barrier” OR “gut epithelial barrier” OR “epithelial barrier” OR “mucosal integrity” OR “intestinal integrity” OR “gut integrity” OR “epithelial integrity” OR “tight junction” OR “intestinal mucosal injury” OR “intestinal epithelial injury” OR “intestinal injury” OR “gastrointestinal mucositis” OR “intestinal mucositis” OR “bacterial translocation” OR endotoxemia OR endotoxaemia OR “intestinal adaptation” OR “intestinal morphology” OR “mucosal morphology” OR “I-FABP” OR “intestinal fatty acid binding protein” OR zonulin)	None	167
